# TLR2, TLR4 and CD14 Recognize Venom-Associated Molecular Patterns from *Tityus serrulatus* to Induce Macrophage-Derived Inflammatory Mediators

**DOI:** 10.1371/journal.pone.0088174

**Published:** 2014-02-07

**Authors:** Karina Furlani Zoccal, Claudia da Silva Bitencourt, Francisco Wanderley Garcia Paula-Silva, Carlos Artério Sorgi, Karla de Castro Figueiredo Bordon, Eliane Candiani Arantes, Lúcia Helena Faccioli

**Affiliations:** 1 Departamento de Análises Clínicas, Toxicológicas e Bromatológicas, Faculdade de Ciências Farmacêuticas de Ribeirão Preto, Universidade de São Paulo, Ribeirão Preto, São Paulo, Brazil; 2 Departamento de Física e Química, Faculdade de Ciências Farmacêuticas de Ribeirão Preto, Universidade de São Paulo, Ribeirão Preto, São Paulo, Brazil; Centro de Investigacion y de Estudios Avanzados del Instituto Politecnico Nacional, Mexico

## Abstract

Scorpion sting-induced human envenomation provokes an intense inflammatory reaction. However, the mechanisms behind the recognition of scorpion venom and the induction of mediator release in mammalian cells are unknown. We demonstrated that TLR2, TLR4 and CD14 receptors sense *Tityus serrulatus* venom (TsV) and its major component, toxin 1 (Ts1), to mediate cytokine and lipid mediator production. Additionally, we demonstrated that TsV induces TLR2- and TLR4/MyD88-dependent NF-κB activation and TLR4-dependent and TLR2/MyD88-independent c-Jun activation. Similar to TsV, Ts1 induces MyD88-dependent NF-κB phosphorylation via TLR2 and TLR4 receptors, while c-Jun activation is dependent on neither TLR2 nor TLR4/MyD88. Therefore, we propose the term venom-associated molecular pattern (VAMP) to refer to molecules that are introduced into the host by stings and are recognized by PRRs, resulting in inflammation.

## Introduction

Human envenomation caused by scorpion stings is a serious public health issue worldwide. The recognition of scorpion venom by mammalian cells triggers a strong inflammatory response that is characterized by increased levels of local and circulating leukocytes [1], [2] and that, in severe cases, can cause pulmonary edema and death in children [3].

*Tityus serrulatus* venom (TsV) is a complex mixture composed of a wide array of substances. It is composed of insoluble mucus, many neurotoxic proteins that affect Na^+^ or K^+^ channels, proteases, hyaluronidase [4] and other peptides, whose biological functions are still not clarified [5], [6]. Ts1, the major toxin component of TsV, corresponds to 16% of the crude soluble venom and contributes significantly to venom toxicity [7]. Ts1 (Uniprot ID P15226, PDB 1NPI and 1B7D) contains 61 amino acid residues, including 8 cysteine residues. Ts1 has a theoretical molecular mass of 6,890.9 Da and a pI of 8.67 (http://web.expasy.org/protparam). Ts1 is a sodium channel β-neurotoxin, which depolarizes the membrane, reduces the amplitude of the action potential and increases its duration [8]. The structure of Ts1 is composed of a highly conserved dense core formed by three anti-parallel β-strands and a β-helix that is interlinked by disulfide bridges [9].

It has previously been reported that interleukin-1α (IL-1α), tumor necrosis factor-α (TNF-α,) interleukin-6 (IL-6), interferon-γ (INF-γ), granulocyte macrophage colony-stimulating factor (GM-CSF), interleukin-10 (IL-10), leukotriene B_4_ (LTB_4_), and prostaglandin E_2_ (PGE_2_) are the primary mediators induced by TsV and its purified toxins, Ts1, Ts2 and Ts6 [1], [2], [10], [11], [12], [13]. Data from the literature have demonstrated that inflammatory mediators are also released during inflammatory responses that are induced as the result of the recognition of either pathogen-associated molecular patterns (PAMPs) or damage-associated molecular patterns (DAMPs) by pattern recognition receptors (PRRs) on innate immune cells [14], [15], [16], [17], [18]. Toll-like receptors (TLRs) are the PRRs of the innate immune system that sense pathogen invasion, can distinguish microbial patterns that are distinct from host molecules [19] and detect non-microbial signal indicators of tissue injury [20] with high sensitivity and specificity. TLRs are expressed on a variety of leukocytes but are particularly abundant on macrophages [21]. Pattern recognition by TLRs activates signaling pathways that result in an inflammatory response. Multiple events are induced during this process, including the phosphorylation of the proteins NF-κB and transcription factor activator protein 1 (AP-1), which translocate into the nucleus and induce the expression of inflammatory genes. In unstimulated cells, the canonical NF-kB dimeric protein, composed of the p50 and p65 subunits, is bound to the NF-kB inhibitor-α (Iκ-Bα) in the cytoplasm. Upon stimulation with endogenous inducers of NF-κB, such as IL-1β and TNF-α, or potent exogenous inducers, such as lipopolysaccharide (LPS), IκBα is rapidly phosphorylated by IκB kinases (IKK) and marked for ubiquitination and degradation in the cytoplasm. The released NF-κB dimer can then be activated by p65 phosphorylation and can translocate into the nucleus, where NF-κB triggers the transcription of target genes by binding to κB elements in the gene promoter regions with high affinity [22], [23], [24]. AP-1, a heterodimer composed of proteins belonging to c-Fos, c-Jun, ATF and JDP families, is also involved in the inflammatory response [25]. Upon stimulation, TLRs activate complex signaling cascades, such as the mitogen-activated protein kinase (MAPK) pathway, which includes the extracellular-signal-regulated kinase (ERK), the c-Jun N-terminal kinase (JNK) and p38. The phosphorylation of AP-1 induces the transcription of TNF-α, IL-1β and matrix metalloproteinase [25], [26]. CD14 is a cell surface receptor that cooperates with TLR4 and MD2 to mediate the innate immune response to LPS in macrophages [27], [28], [29]. Downstream of the tripartite receptor, the recruitment of the adaptor proteins TIR-containing adaptor molecule (TIRAP) and myeloid differentiation factor (MyD88) initiates a MyD88-dependent pathway that culminates in the activation of the NF-κB and MAPK pathways [30]. In addition to the MyD88-dependent pathway, LPS stimulation also results in the activation of a MyD88-independent pathway through the recruitment of the adaptor molecules TIR-containing adaptor inducing IFN-β (TRIF) and TRIF-related adaptor molecule (TRAF). This recruitment leads to the late-phase activation of NF-κB, interferon regulatory factor 3 (IRF3), MAPKs and phosphatidylinositol 3-kinase (PI3K) [31].

Currently, the mechanisms underlying the innate immune recognition and the cell signaling networks that are involved in the inflammatory response following envenomation by scorpions are unknown. Using knockout mice, we identified, for the first time, the innate immune receptors and cell signaling pathways involved in macrophage activation by TsV and Ts1. In the present manuscript, we describe, for the first time, a role for TLR2, TLR4 and CD14 in the innate immune recognition of TsV and purified Ts1, which we named venom-associated molecular patterns (VAMPs). Additionally, we identified the macrophage signaling pathways involved in TNF-α, IL-6, PGE_2_ and LTB_4_ production following VAMP recognition. Specifically, our results demonstrated that TsV interacts with TLR2, TLR4 and CD14. This interaction recruits MyD88 and activates the AP-1 and NF-κB pathways. Similarly, Ts1 induces TNF-α and IL-6 production in a manner that is dependent on TLR2, TLR4 and CD14, although signaling occurs specifically through NF-κB activation.

## Materials and Methods

### Animals

TLR2, TLR4, CD14and MyD88 knockout (KO or ^−/−^) mice between the ages of 6 and 8 weeks were donated by S. Akira (Osaka University, Osaka, Japan) and bred in the Animal House of the Faculdade de Medicina de Ribeirão Prêto (Universidade de São Paulo, Ribeirão Prêto, Brazil). Strain-matched WT C57Bl/6 (C57Bl/6 was the genetic background for the TLR2, TLR4, CD14 and MyD88 KO mouse) mice of both sexes were bred in the Faculdade de Ciências Farmacêuticas de Ribeirão Prêto (Universidade de São Paulo, Ribeirão Preto, Brazil). The mice were maintained in a room at 25°C, with a 12 h/12 h light/dark cycle, and provided with free access to food and water. The mice were kept in biohazard facilities. All experiments were approved and conducted in accordance with the guidelines of the Animal Care Committee of Prefeitura of Campus of Ribeirão Preto (PCARP) at the University of São Paulo (Protocol number 11.1.160.53.1).

### Toxins

TsV was extracted from *Tityus serrulatus*, and the Ts1 toxin, representing 16% of the total crude soluble TsV, was purified and stored at −20°C, as previously described [32]. Prior to the experiments, TsV and Ts1 were dissolved in phosphate buffered saline (PBS) and filtered through 0.22 µm sterilizing membranes. To determine whether the purified toxins were contaminated with LPS, a *Limulus amoebocyte lysate* (LAL) test was performed (QCL-1000, Bio Whittaker, Cambrex Company, East Rutherford, NY, USA).

### Macrophage Culture and Activation

The macrophages were isolated from the peritoneal cavities of mice and plated in 96-well micro culture plates at a density of 2×10^5^ cells/well in RPMI medium supplemented with 10 mM L-glutamine, 100 U/ml penicillin, 100 U/ml streptomycin and 10% fetal bovine serum (FBS). The cells were cultured at 37°C in a humidified 5% CO_2_ atmosphere for 18 h. After this period, the supernatants were collected and the cells were stimulated either with TsV (50 µg/ml/2×10^5^ cells) for 30 min or 24 h or with Ts1 (50 µg/ml/2×10^5^ cells) for 24 h at 37°C in a 5% CO_2_ atmosphere. After the stimulation period, the culture supernatants were harvested and stored at −20°C until further use. To verify whether the recognition of TsV or Ts1 by macrophages was dependent on their tertiary structures, TsV and Ts1 were thermally inactivated prior to being added to the cell cultures. For heat inactivation, 50 µg/mL each of TsV and Ts1 were heated for 30 min at 60°C [33], and for heat denaturation, TsV and Ts1 were boiled at 95°C for 60 min. The supernatants were collected for IL-6 and TNF-α measurement by ELISA, as described below.

### Assessment of Cell Injury

To ensure the stimuli were not cytotoxic, lactate dehydrogenase (LDH) levels in the macrophage supernatants were measured after 24 h of incubation with TsV or Ts1. The level of LDH in the cell supernatant was measured using the CytoTox96® non-radioactive assay (Promega) at 490 nm. LDH levels were expressed as percentages of the LDH levels observed in control cultures.

### Cytokine Measurements

The supernatants from the cells stimulated with TsV or Ts1 were used to measure IL-6 and TNF-α levels by enzyme-linked immunosorbent assays (ELISA), with specific purified and biotinylated antibodies and cytokine standards, according to the manufacturer’s instructions (R&D Systems). The optical densities were measured at 450 nm in a micro plate reader (µQuant, Biotek Instruments Inc.). For each sample, the cytokine levels were obtained from a standard curve established with the appropriate recombinant cytokine. The results are expressed as pg/mg of total protein. The sensitivities were >10 pg/ml.

### LTB_4_ and PGE_2_ Measurements

A specific enzyme immunoassay (Cayman Chemical) was used to quantify LTB_4_ and PGE_2_ in the supernatants of cells stimulated with TsV or Ts1, according to the manufacturer’s instructions. The sample absorbance was measured at 420 nm in a micro plate reader (µQuant, Biotek Instruments Inc.), and the concentrations of eicosanoids were calculated based on the standard curve. The detection limit was >13 pg/ml.

### Gene Expression Analysis by qRT-PCR

mRNA expression was evaluated after 4 h of macrophage stimulation with TsV or Ts1 using a custom RT_2_ Profiler PCR Array kit (Qiagen Inc.). Total RNA was isolated using the RNeasy Mini kit (Qiagen Inc.), and the reverse transcription of 500 ng of RNA was performed using the RT_2_ HT First Strand kit (Qiagen Inc.). Ten nanograms of the cDNA template was used per reaction, and the reactions were performed with specific pre-designed primers for *Tlr2* (Toll-like receptor 2), *Tlr4* (Toll-like receptor 4), *Cd14* (CD 14 molecule), *Myd88* (Myeloid differentiation primary response gene 88), *Ltb4r1* (leukotriene B_4_ receptor 1), *Alox5* (Arachidonate 5-Lipoxygenase), *Alox5ap* (Arachidonate 5-Lipoxygenase-Activating Protein), *Ptgs2* (Prostaglandin-Endoperoxide Synthase 2 or Cyclooxygenase-2) and *Ptges2* (Prostaglandin E synthase 2). The endogenous internal controls used were *Tbp* (Tubulin), *Actb* (Beta actin) and *Gapdh* (Glyceraldehyde 3-phosphate dehydrogenase). Amplification was performed in an Eppendorf Mastercycler**®** ep realplex 4 (Eppendorf AG) using the SYBR Green Mastermix. Denaturation of the DNA was followed by 40 cycles of 95°C for 15 s and 60°C for 1 min. The 2^–ΔΔCt^ method was used for the analysis of the RT-PCR data.

### NF-κB Reporter Assay

RAW-Blue™ cells, RAW264.7 macrophages that stably express the secreted embryonic alkaline phosphatase (SEAP) gene, which is inducible by the NF-κB/AP-1 transcription factors and is resistant to the selectable marker Zeocin, were donated by Huy Ong (Université de Montréal, Canada). The cells were seeded in 96-well micro culture plates at a density of 2×10^5^ cells/well in DMEM supplemented with Normocin™ (50 mg/mL) and cultured at 37°C in a humidified 5% CO_2_ atmosphere for 18 h. After this period, the cells were incubated with 10 ng/ml of LPS from *Rhodobacter sphaeroides*, a TLR4 antagonist (LPS-RS - InvivoGen), with or without LPS from *E. coli* (0.5 µg/ml), and with TsV (50 µg/ml) or Ts1 (50 µg/ml) for 24 h. In another experiment, the cells were pre-incubated with 100 ng/ml of a purified monoclonal IgG antibody against mouse TLR2 (anti-mTLR2-IgG – InvivoGen) for 30 min and then stimulated with LPS from *E. coli* (0.5 µg/ml) and TsV or Ts1 (50 µg/ml) for 24 h. After 24 h of stimulation, the medium was harvested, and 50 µl samples were mixed with QUANTI-Blue™ (InvivoGen), which is a SEAP detection medium (150 µl), in 96-well plates at 37°C for 2 h. The optical density was then measured at 650 nm using an ELISA reader.

### Immunoassay of Phosphoproteins using the Pathscan ELISA kit

To assess the levels of several phosphoproteins that activate inflammation in macrophages, a multi-target ELISA kit was used (PathScan® Inflammation Multi-Target Sandwich ELISA Kit, ref. 7276, Cell Signaling). Peritoneal macrophages were plated in 12-well microculture plates at a density of 3×10^6^ cells/well in RPMI medium and cultured at 37°C in a humidified 5% CO_2_ atmosphere for 18 h. The cells were then stimulated with TsV or Ts1 (both at 50 µg/ml) for 10 min, 2 h or 24 h at 37°C in a 5% CO_2_ atmosphere. After treatment, the adherent macrophages were washed with ice-cold PBS. Then, Cell Lysis Buffer (Cell Signaling) containing protease and phosphatase inhibitors was added to the cells. The cells were scraped and transferred to an Eppendorf tube. The lysates were centrifuged (FANEM) at 10,000 × *g* for 10 min at 4°C, and the supernatant was stored in a new tube at −80°C. The total protein content of the lysates was determined using the Bradford method (Sigma-Aldrich). The cell lysates were analyzed, and the proteins were semi-quantified using the PathScan® phospho-NFκBp65 (Ser536), phospho-IκBα (Ser32) and phospho-c-Jun sandwich ELISA kits, according to the manufacturer’s instructions (Cell Signaling Technology). Briefly, the lysates were diluted with sample diluent, and 100 µl of the lysate was added to wells that had been pre-coated with the primary antibody. The plate was incubated overnight at 4°C and then washed 4 times. After washing, the plates were incubated with the detection antibody for 1 h at 37°C. The plates were washed and then incubated with a horseradish peroxidase (HRP)-conjugated secondary antibody for 30 min at 37°C. The plates were then incubated with a 3,3′,5,5′-tetramethylbenzidine (TMB) substrate for 30 min at room temperature. Finally, the reaction was stopped, and the absorbance of the samples was read at 450 nm. The assay was performed in technical duplicates. The results were expressed as percentages of the levels of phosphorylated proteins in the control.

### Cytometric Bead Array Flex Set for MAPKs

The sample preparation was performed according to the manufacturer’s protocol for adherent cells (Becton Dickinson). Peritoneal macrophages were plated in 12-well microculture plates as described above. The cells were stimulated with TsV (50 µg/ml) for 15, 30, 60 and 120 min at 37°C in a 5% CO_2_ atmosphere. RPMI medium was used as the negative control. After treatment, the adherent macrophages were washed with ice-cold PBS, and Cell Lysis Buffer containing protease and phosphatase inhibitors was added. The cell lysates were transferred to an Eppendorf tube and immediately placed in a boiling water bath for 5 min. The total protein concentration was adjusted to 1 µg/µl, and the cell lysates were stored at −80°C prior to measurement. The expression levels of p-JNK1/2 (T183/Y185), p-p38 (T180/Y182) and p-ERK1/2 (T202/Y204) were quantitatively measured using antibodies from the multiplex Flex Set Cytometric Bead Array (Becton Dickinson). Serial dilutions (1/2 v/v) of the standards were prepared, and the cell lysates were diluted (1/4 v/v) using the assay diluent. The samples were incubated with 50 µl of the mixed capture beads for 3 h at room temperature, followed by incubation with 50 µl of phycoerythrin (PE) detection reagent for 1 h at room temperature. Then, 300 µl of wash buffer was added, and the samples were centrifuged at 400 × *g* for 5 min. Flow cytometry was performed using the Becton Dickinson FACSArray Bioanalyzer software. A total of 900 events were acquired, according to the manufacturer’s protocol. The minimum detection levels for each phosphoprotein were 0.38 U/ml for p-JNK and 0.64 U/ml for p-p38 and p-ERK.

### Statistical Analyses

The data are expressed as the means ± SEM and were analyzed using one-way ANOVA (α = 0.05). *p* values lower than 0.05 were considered statistically significant. For gene expression, changes in expression levels were considered statistically significant when *p*<0.05 and a fold-change >2.0 was detected.

## Results

### TsV and Ts1 Induce the Production of Cytokines and Lipid Mediators without Affecting Cell Viability

**Figure 1:**
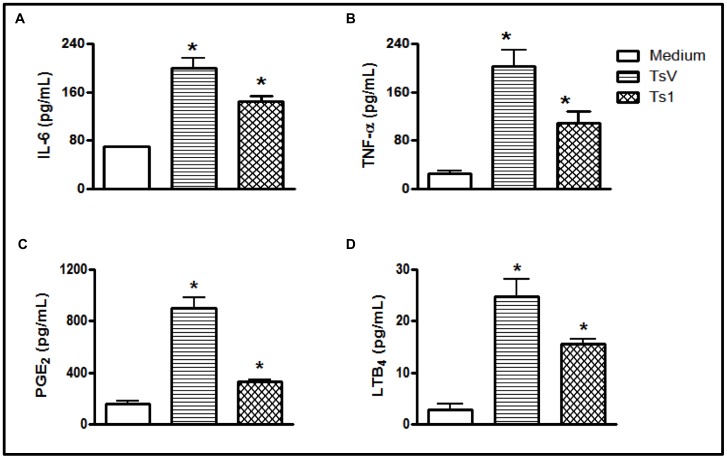
TsV and Ts1 induce IL-6, TNF-α, PGE_2_ and LTB_4_ production in peritoneal macrophages. Adherent macrophages from C57Bl/6 (WT) mice were stimulated with TsV or Ts1 (50 µg/ml), and the supernatants were collected after 24 h, following incubation in a 5% CO_2_ atmosphere at 37°C. The levels of IL-6 (a), TNF-α (b), PGE_2_ (c) and LTB_4_ (d) in the supernatants were measured by ELISA. Medium alone was used as the negative control. *p<0.05 (one-way ANOVA) compared to medium alone. The data from 3 independent experiments are shown (*n* = 12).

To investigate the effects of TsV on the viability of peritoneal macrophages obtained from C57Bl/6 mice, macrophages were exposed to TsV for 24 h. The concentrations (25–150 µg/ml) of TsV used in these experiments had no cytotoxic effects (Fig. S1A). We investigated the absence of cell injury induced by TsV and Ts1 by measuring LDH release in macrophage supernatants. We observed that the incubation of cells for 24 hours with TsV (50 µg/ml) or Ts1 (50 µg/ml) did not induce membrane disruption, unlike the LDH positive control (from the kit) (Fig. S1B). We then investigated the capacity of TsV and Ts1 to induce IL-6 and TNF-α production in peritoneal macrophages. As shown in Fig. 1A and B, after 24 h of stimulation, both TsV and Ts1 provoked increases in the concentrations of IL-6 and TNF-α in the cell supernatants when compared to medium alone. Interestingly, significant reductions in IL-6 or TNF-α release were observed in macrophages stimulated with either TsV or Ts1 that was heated to 60°C prior to being added to the culture medium, and the total inhibition of cytokine production was observed when TsV and Ts1 were heated to 95°C prior to being added to the culture medium (Figs. S1C and S1D). Together these results demonstrate that cytokine induction by TsV or Ts1 is dependent on their tertiary structure recognition by PRRs. Finally, we confirmed that the cytokine induction observed following the addition of the venom to the cell cultures was not due to the presence of LPS in the venom preparations. Using the *Limulus* amoebocyte lysate test (data not shown) and TsV polymyxin B treatment (a method used to neutralize contaminating LPS), we observed no changes in TNF-α induction (Fig. S2).

Lipid mediators are involved in inflammation, the regulation of the immune response and several homeostatic biological functions [34], [35], [36], [37], [38]. Additionally, LTB_4_ and PGE_2_ are produced in response to Ts2 and Ts6, which are toxins that can be isolated from scorpion venom [2], and PGE_2_ participates in TsV-induced edematous responses in mice [39]. To determine whether TsV and Ts1 also induce PGE_2_ and LTB_4_ production in peritoneal macrophages, we measured the concentration of these lipid mediators in the cell culture supernatants after 24 h of stimulation. We found that both TsV and Ts1 significantly induced PGE_2_ and LTB_4_ release in peritoneal macrophages (Fig. 1C and Fig. 1D).

### TsV and Ts1 Signaling occurs through TLR2, TLR4 and CD14

**Figure 2:**
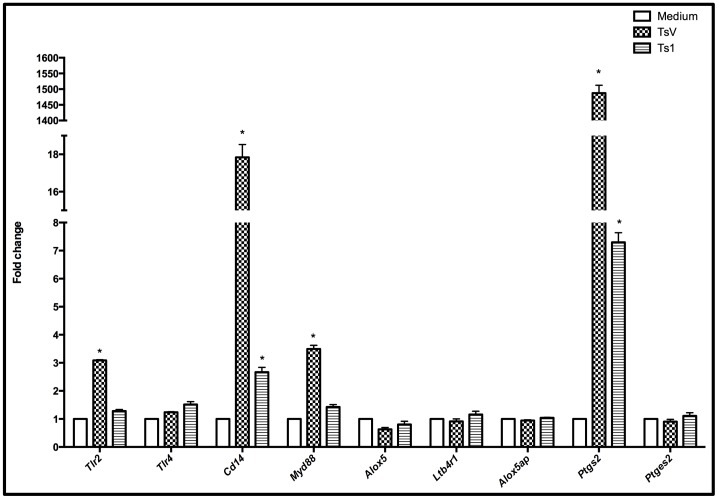
TsV and Ts1 stimulation increases the mRNA expression of *Tlr2, Cd14, Myd88* and *Ptgs2* in peritoneal macrophages. Adherent macrophages from C57Bl/6 (WT) mice were treated with TsV or Ts1 (50 µg/ml) for 4 h. Unstimulated macrophages were used as the negative control. The cells were lysed, and total RNA was extracted. qRT-PCR was performed to determine the relative expression levels of transcripts encoding lipid metabolism enzymes, TLRs and adaptor proteins. The results were normalized to the expression levels of the endogenous internal controls *Actb*, *Gapdh* and *Tbp*. The 2^–ΔΔCt^ method was used for the analysis of the qRT-PCR data. *p<0.05 (one-way ANOVA followed by Dunnett’s post-test) compared to medium alone. Statistically significant changes were considered when *p*<0.05 and any gene presented a fold-change >2.0. The results are presented as the fold-change measured from 2 independent experiments.

Because of the increased production of the inflammatory mediators TNF-α, IL-6, PGE_2_ and LTB_4_ following TsV and Ts1 stimulation, we evaluated the gene expression of potential PRRs, adaptor molecules and enzymes involved with cytokines and arachidonic acid metabolism. For these studies, the macrophages were cultured with or without TsV or Ts1 for 4 h. Quantitative real time polymerase chain reaction (qRT-PCR) analysis of stimulated and non-stimulated macrophages revealed that TsV up-regulated the expression levels of the genes *Tlr2, Cd14, Myd88* and *Ptgs2* without affecting the expression levels of *Tlr4, Alox5ap, Ltb4r1* or *Ptges2.* Furthermore, Ts1 up-regulated the *Cd14* and *Ptgs2* genes without affecting the expression levels of *Tlr2, Tlr4, Myd88, Alox5ap, Ltb4r1* and *Ptges2.* The effects of Ts1 on gene expression were less dramatic than those observed with TsV-stimulation (Fig. 2).

**Figure 3:**
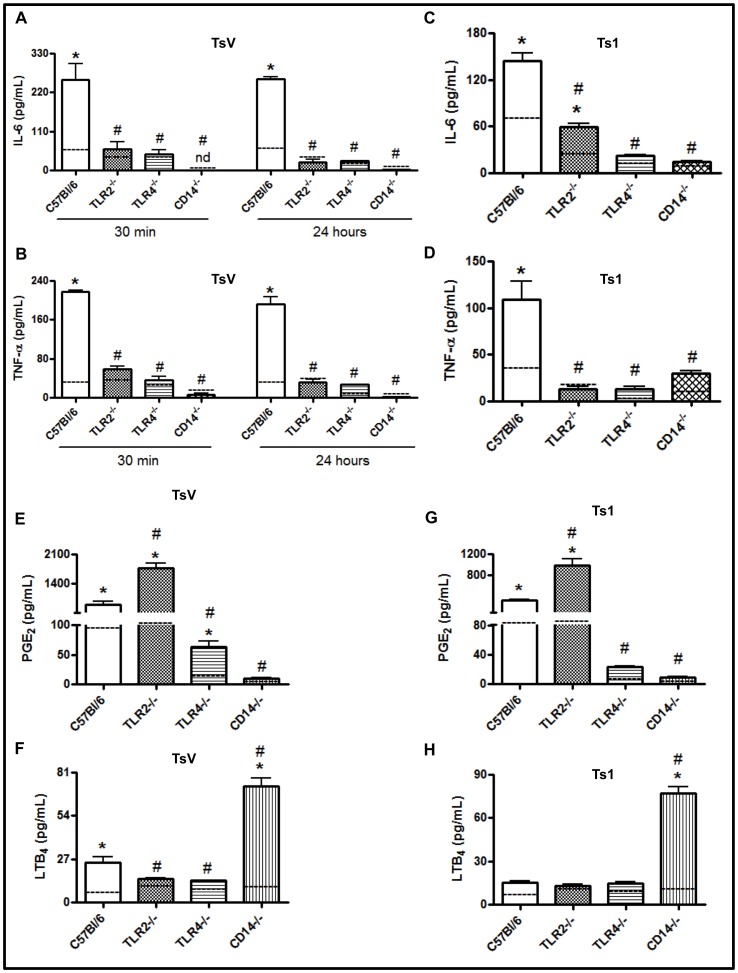
TLR4, TLR2 and CD14 mediate the recognition of TsV and Ts1 and modulate IL-6, TNF-α, PGE_2_ and LTB_4_ production. Peritoneal macrophages from C57Bl/6 (WT) mice, TLR2^−/−^, TLR4^−/−^ or CD14^−/−^ mice were stimulated with TsV (50 µg/ml) (a, b) for 30 min or 24 h or with TsV (e, f) or Ts1 (50 µg/ml) (c, d, g, h) for 24 h in a 5% CO_2_ atmosphere at 37°C. The concentrations of IL-6 (a, c), TNF-α (b, d), PGE_2_ (e, g) and LTB_4_ (f, h) in the culture supernatants were determined by ELISA. *p<0.05 (one-way ANOVA) compared to the WT mice. The values represent the means ± SD (*n* = 8), and the data are from 2 independent experiments.

Since TsV induced IL-6 and TNF-α production in peritoneal macrophages and up-regulated the expression levels of the *Tlr2*, *Cd14* and *Myd88* genes, we then determined whether the TLR2, TLR4 and CD14 signaling pathways were involved in TsV-induced cellular activation. We observed that 30 min or 24 h following TsV-stimulation, the IL-6 (Fig. 3A) and TNF-α (Fig. 3B) concentrations were significantly decreased in the supernatants of TLR2-, CD14- or TLR4- knockout (KO) peritoneal macrophages when compared to wild-type (WT) cells. Similarly, peritoneal macrophages obtained from TLR2-, CD14- or TLR4-KO mice that were stimulated with Ts1 (50 µg/ml/24 hours) produced less IL-6 (Fig. 3C) and TNF-α (Fig. 3D) than macrophages from WT C57Bl/6 mice. Together, these data indicate that the TsV-induced inflammatory cytokine release is dependent on TLR4, TLR2 and CD14 molecules and suggest that Ts1 is the primary component involved in the cytokine response to TsV.

Recently, we demonstrated that Ts2 and Ts6 induced inflammation through mechanisms that were dependent on cytokine and lipid mediator generation [2]. Therefore, we next determined whether the LTB_4_ and PGE_2_ induction caused by TsV or Ts1 in macrophages was dependent on the same receptors involved in cytokine production. Compared to medium alone (control), we observed the increased production of both LTB_4_ and PGE_2_ following WT macrophage stimulation with either TsV or Ts1. On the contrary, the PGE_2_ production in response to TsV or Ts1 was almost completely absent in TLR4- and CD14-KO cells. Intriguingly, the amount of PGE_2_ produced by cells lacking TLR2 was significantly higher than the quantity released by WT cells (Figs. 3E and 3G). In contrast, the LTB_4_ production induced by TsV was partially dependent on TLR2 and TLR4 (Fig. 3F), and LTB_4_ production was independent of TLR2 and TLR4 upon stimulation with Ts1 (Fig. 3H). Surprisingly, higher LTB_4_ concentrations were detected in the supernatants of CD14-KO macrophages. Our results indicate that TLR2 and CD14 receptors are involved in the negative regulation of PGE_2_ and LTB_4_ production, respectively, in response to TsV and Ts1.

### Cell Signaling Pathway Activation following TsV Recognition

The MAPKs p38, ERK1/2 and JNK1/2 are activated by specific TLRs [26] and their ligands [40], inducing the production of inflammatory cytokines and lipid mediators [41]. To identify the signaling pathway involved in TsV-induced cellular activation, we stimulated C57Bl/6 peritoneal macrophages with TsV for 10, 30, 60 and 120 minutes. TsV (50 µg/ml) treatment led to p38 and ERK1/2 phosphorylation but did not alter the phosphorylation state of JNK1/2 (Fig. S3).

The activation of NF-κB following TLR stimulation in monocytes and macrophages is also required for the induction of inflammatory mediators [42]. We analyzed the proteins involved in cell signaling pathways triggered by TsV that induced IL-6, TNF-α, PGE_2_ and LTB_4_ production and discovered that TsV induced an increase in the phosphorylation of IκB-α, NF-κB and c-Jun; c-Jun is a transcription factor downstream of the MAPKs (Fig. S4). These data indicate that TsV recognition induces the phosphorylation of proteins that are involved in the activation of the MAPK and NF-κB pro-inflammatory pathways.

**Figure 4:**
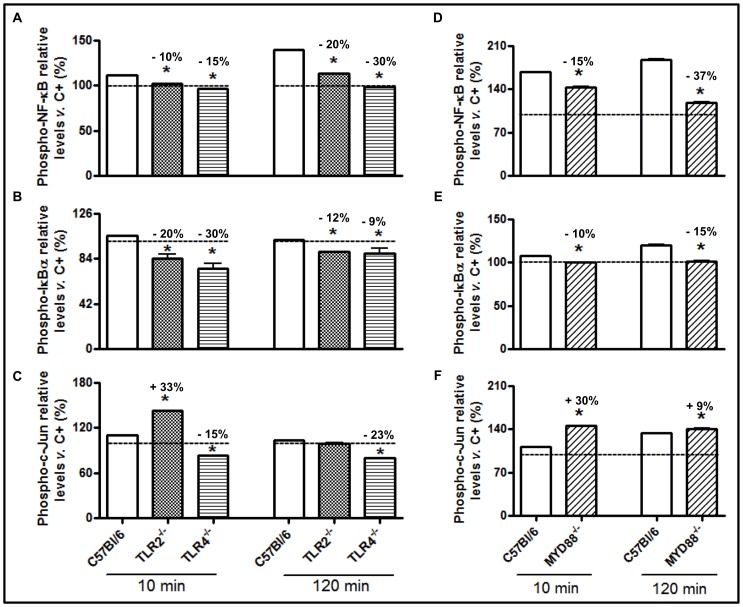
TsV induces TLR2- and TLR4/MyD88-dependent activation of NF-κB and TLR2/MyD88-independent and TLR4-dependent activation of AP-1. Adherent peritoneal macrophages from WT (C57Bl/6), TLR2^−/−^, TLR4^−/−^ or MyD88^−/−^ mice were stimulated with TsV (50 µg/mL) for 10 or 120 min in a 5% CO_2_ atmosphere at 37°C. The p-NF-κB (a, d), p-IκBα (b, e) and p-c-Jun (c, f) protein levels were determined using the PathScan Inflammation Multi-Target Sandwich ELISA kit. The results are presented as a percentage of the phosphoprotein level in non-stimulated control cell lysate (dashed line). *p<0.05 (one-way ANOVA) compared to WT. The values represent the means ± SD (*n* = 4), and the data are from 2 independent experiments.

Our next objective was to determine whether the activation of the NF-κB and c-Jun inflammatory pathways was dependent on the interactions between TsV and TLR2 and TLR4 or on signaling via the adaptor molecule MyD88. TsV induced NF-κB (Fig. 4A) and IκBα (Fig. 4B) phosphorylation in C57Bl/6 (WT) cells. In contrast, the levels of phosphorylated NF-κB and IκBα in peritoneal macrophages from TLR2^−/−^ and TLR4^−/−^ mice were lower than those observed in WT cells. However, early and increased c-Jun phosphorylation was observed in TLR2^−/−^ peritoneal macrophages stimulated with TsV, unlike TLR4^−/−^ cells (Fig. 4C). Together, these results demonstrate that TsV-induced NF-κB activation requires TLR2/TLR4, whereas c-Jun phosphorylation is dependent on TLR4 only.

**Figure 5:**
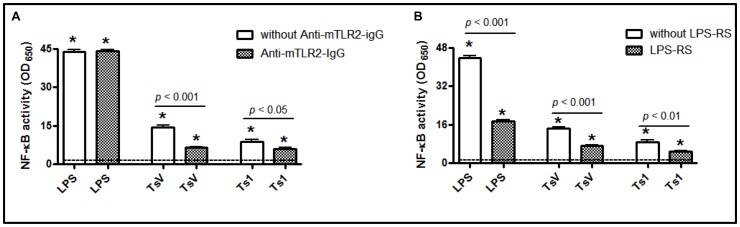
Activation of NF-κB/AP-1 in RAW-Blue™ cells. These cells were derived from RAW 264.7 macrophages and contain a secreted embryonic alkaline phosphatase (SEAP) reporter construct that is integrated into the cellular DNA and that can be induced by NF-κB. The cells were incubated with either (A) anti-mTLR2-IgG (100 ng/ml) or (B) LPS-RS (10 ng/ml) for 30 min, with or without LPS (0.5 µg/ml), and TsV or Ts1 (50 µg/ml) for 24 h. The QUANTI-Blue™ substrate was used to measure the SEAP at 650 nm with an ELISA reader. The measurements were performed in triplicate, and a representative experiment is shown. *p<0.05 (one-way ANOVA) compared to medium alone (dashed line). The values represent the means ± SD (*n* = 8), and the data are from 2 independent experiments.

The adaptor molecule MyD88 forms homodimers to promote the recruitment and activation of IL-1 receptor-associated IRAK-1 and IRAK-4, which is followed by the activation of the inhibitory κB kinase (IKK) and the mitogen-activated protein kinase (MAPK) [43]. These kinases are pivotal for the activation of several transcription factors, including AP-1 and NF-κB. In the present study, we determined whether the MyD88 adaptor molecule was involved in TsV-induced cell signaling. As shown in Fig. 4, phosphorylated NF-κB (Fig. 4D) and IκBα (Fig. 4E) levels in MyD88^−/−^ peritoneal macrophages were lower than those observed in C57Bl/6 macrophages after 10 or 120 min of stimulation with TsV. However, c-Jun phosphorylation was increased after 10 min of TsV-stimulation, with no differences being observed after 120 min (Fig. 4F). Additionally, we confirmed that TsV and Ts1 induce NF-κB activation using another experimental approach. Our results demonstrated that the treatment of RAW-Blue™ cells with a TLR4 antagonist (LPS-RS) or a TLR2 receptor blocker (anti-mTLR2-IgG) prior to the addition of TsV or Ts1 significantly inhibited the secretion of embryonic alkaline phosphatase when compared with cells treated with TsV or Ts1 alone (Fig. 5). LPS, a TLR4 agonist that activates the NF-κB pathway, was used as a positive control.

### The Receptors Involved in Ts1-induced Intracellular Signaling are Similar to the Receptors Activated in Response to TsV

**Figure 6:**
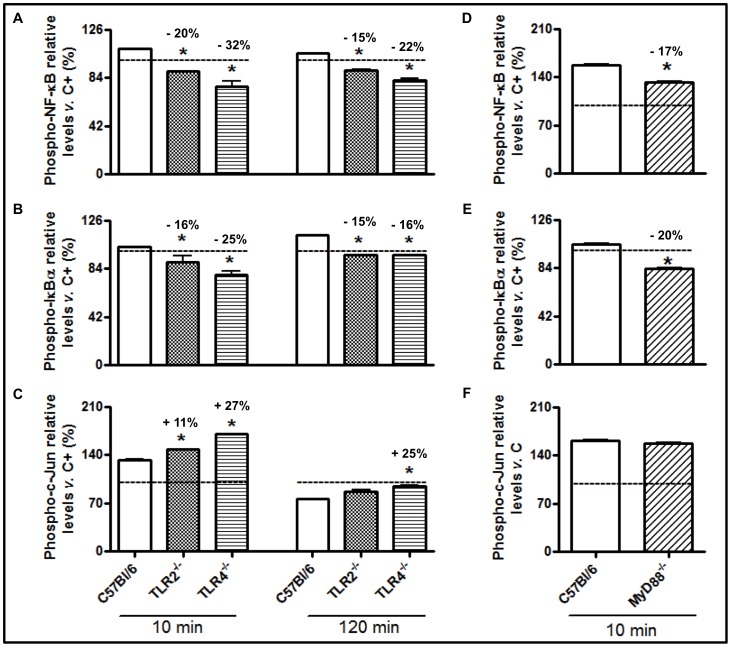
Ts1 induces TLR2- or TLR4/MyD88-dependent activation of NF-κB and TLR2- or TLR4/MyD88-independent activation of AP-1 in stimulated macrophages. Adherent peritoneal macrophages from WT (C57Bl/6), TLR2^−/−^, TLR4^−/−^ or MYD88^−/−^ mice were stimulated with Ts1 (50 µg/ml) for 10 or 120 min in a 5% CO_2_ atmosphere at 37°C. The p-NF-κB (a, d), p-IκBα (b, e) and p-c-Jun (c, f) protein levels were determined using the PathScan Inflammation Multi-Target Sandwich ELISA kit, as described in the Materials and Methods section. The results are presented as a percentage of the phosphoprotein level in non-stimulated control cell lysate (dashed line). *p<0.05 (one-way ANOVA) compared to WT. The values represent the means ± SD (*n* = 4), and the data are from 2 independent experiments.

Next, we determined whether the Ts1-induced activation of intracellular signaling was similar to that induced by TsV. Peritoneal macrophages from C57BL/6, TLR2^−/−^ and TLR4^−/−^ mice were stimulated *in vitro* with Ts1 for 10 or 120 min. We observed increased levels of p-NF-κB (Fig. 6A), p-IκBα (Fig. 6B) and p-c-Jun (Fig. 6C) in C57BL/6 peritoneal macrophages, whereas decreases in the phosphorylation of NF-κB and IκBα in TLR2^−/−^ and TLR4^−/−^ peritoneal macrophages were detected. Similar to what was observed with TsV stimulation, we also detected an increase in the phosphorylation of c-Jun in TLR2^−/−^ and TLR4^−/−^ peritoneal macrophages stimulated with Ts1, compared to WT macrophages (Fig. 6C).

**Figure 7:**
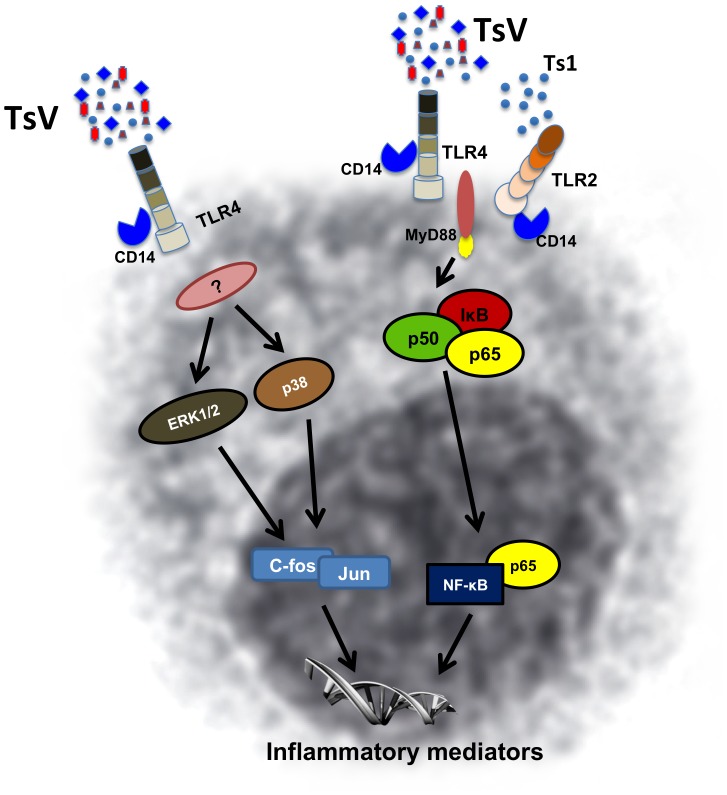
A schematic diagram showing the increased pro-inflammatory cytokine production in peritoneal macrophages stimulated with TsV and Ts1. Pro-inflammatory cytokine production occurs via the following two routes: (1) MyD88-dependent signaling, where TsV and Ts1 are recognized by TLR4/CD14/TLR2, resulting in NF-kB nuclear translocation; and (2) MyD88-independent signaling, where TsV is recognized by TLR4/CD14 and activates ERK1/2 and p38 phosphorylation and c-Fos/Jun expression.

Peritoneal macrophages from MyD88^−/−^ mice that were stimulated with Ts1 (50 µg/ml) for 10 min had decreased levels of phosphorylation for NF-κB (Fig. 6D) and IκBα (Fig. 6E) compared to the WT macrophages. Nonetheless, c-Jun phosphorylation in MyD88^−/−^ cells was similar to WT macrophages (Fig. 6F). Altogether, our data indicate that TsV and Ts1 are TLR2, TLR4 or CD14 agonists, competent to trigger intracellular signaling pathways, resulting in MyD88 dependent NF-κB phosphorylation and MyD88 independent c-Jun activation (Fig. 7).

## Discussion

Sensing danger is critical for the initiation of the innate immune response and the preservation of host integrity. Cells responsible for the recognition of PAMPs and DAMPs express PRRs, and several molecules that provoke cell signaling, leading to the release of inflammatory mediators, have been identified [44], [45]. However, studies concerning the recognition of scorpion venom and its toxins and their influence on the innate immune response following envenomation are scarce. In the present study, we identified TLR2, TLR4, and CD14 as the receptors that sense TsV and its major component, the Ts1 toxin. We also demonstrated that the recognition of TsV and Ts1 by PRRs was dependent on the tertiary structure of TsV and Ts1 and independent of pathogens or cell-damage-derived products. Therefore, we proposed the term VAMP to refer to molecules that are injected in the host by stings and are recognized by PRRs, resulting in inflammation. We further identified both MyD88-dependent and MyD88-independent pathways downstream from PRRs that are responsible for NF-kB and AP-1 activation and TNF-α, IL-6, PGE_2_ and LTB_4_ release.

Macrophages play a key role in inflammation [46]. These cells express PRRs that recognize DAMPs and PAMPs and release cytokines and lipid mediators [18], [46]. In the present study, we demonstrated that TsV and Ts1 interact with PRRs present in murine macrophages, stimulating inflammatory cytokines and lipid mediator production, independent of cell death. We also demonstrated, for the first time, that TsV- and Ts1-induced TNF-α and IL-6 release was TLR2-, TLR4- and CD14-dependent. Data from the literature demonstrated that TLR2 and TLR4 may function as LPS receptors and that the co-expression of membrane CD14 with TLR4 may preferentially enhance the capacity of TLR4 to confer LPS responsiveness. An additional explanation for these differences may be the recent finding that an additional protein, MD-2, is required to confer LPS responsiveness in cells that express TLR4 [47]. Furthermore, TLR2 recognizes bacterial components, such as peptidoglycan (PGN), bacterial triacylated lipoprotein (Pam_3_CSK_4_), mycoplasma diacylated lipoprotein (Malp2), lipoarabinomannan (AraLAM), zymosan and protozoan GPI anchors [48], [49], [50]. Therefore, our results suggest that TLR2 and TLR4 are important for initiating the inflammation that occurs during envenomation, and CD14 helps to enhance the capacity of that response. However, the details of the subsequent cellular activation pathway still require elucidation.

Moreover, our results demonstrated that PGE_2_ production following TsV or Ts1 stimulation was dependent on TLR4 and CD14, but independent of TLR2, similar to what has been reported for LPS-stimulated macrophages [51]. However, LTB_4_ production following TsV or Ts1 stimulation was dependent on TLR2 and TLR4, but independent of CD14. We have two possible explanations for these results: 1) in the absence of TLR2, the activation of cyclooxygenase-2 is enhanced; or 2) the half-life of cyclooxygenase-2 is enhanced, which is a common and potent negative regulatory mechanism. Regarding the CD14/LTB4 axis, we speculate that, in the absence of CD14, neither TLR4 nor TLR2 act properly. Coffey et al. [52] have shown that LPS inhibits 5-LO activity in a manner that is dependent on the generation of nitric oxide (NO). NO is highly produced during TLR activation, and it has been shown to induce the nitrosylation of 5-LO, impairing the activity of 5-LO. The nature of the binding between the venom and CD14 or any other receptor is not yet known and should be further investigated.

Another objective of the current study was to identify the signaling pathway triggered by TsV downstream of TLR2, TLR4 and CD14. The activation of NF-κB is essential for the coordination of innate and adaptive immune responses, including the production of pro-inflammatory mediators [53], [54]. The data from the present study indicated that TsV and Ts1 stimulation increased the levels of phosphorylated NF-κB and IκBα. These results are consistent with a previous study indicating that, upon LPS stimulation, the IκB proteins are phosphorylated and degraded, allowing NF-κB to move to the nucleus and bind the transcriptional-regulatory elements in a nucleotide sequence-specific manner to activate the transcription of certain genes [55]. Additionally, we demonstrated that ERK1/2 and p38 MAPK activation in TsV-stimulated macrophages contributed to the production of inflammatory cytokines. A previous study demonstrated that bee venom and its primary component, melittin, suppressed the LPS-induced activation of NF-κB by blocking the degradation of IκBα and the phosphorylation of JNK [56]. Because the activation of NF-κB can also be regulated by cellular kinases, such as the MAPKs [55], the possibility of crosstalk between these pathways during TsV-induced inflammatory cytokine production cannot be ruled out.

Subsequently, we determined whether the TsV-mediated activation of NF-κB and AP-1 occurred downstream of TLR2 or TLR4 and whether MyD88 played a role in this process. TLR2 often heterodimerizes with TLR1 or TLR6 to respond to many TLR2 ligands [57], [58], [59]. TLR2 cooperates with TLR6 in response to diacylated mycoplasma lipopeptide [60] and associates with TLR1 to recognize triacylated lipopeptides [61]. It has been well established that TLRs can recognize infectious agents by sensing PAMPs and triggering the antimicrobial immune responses of the host [62], [63]; however, emerging evidence indicates that certain molecules can also activate TLRs, such as TLR2-MyD88, independent of TLR1 and TLR6 [64]. We observed that the activation of NF-κB occurred in a MyD88-dependent manner as a result of TLR2 and TLR4 stimulation, suggesting that these receptors are important in the response to TsV. Notably, the activation of the AP-1 pathway is dependent on TLR4 but can be either dependent on [65] or independent [66] of TLR2 and MyD88. Our results are in agreement with a TLR2/MyD88-independent pathogen recognition pathway [66]. Usually, TLR4 signaling via the MyD88 adaptor leads to pro-inflammatory cytokine production [59], [63], [67], [68], [69], [70]. Because TRIF involvement in pro-inflammatory events has been reported [71], [72], [73], its role in TsV-mediated signaling cannot be discarded.

To the best of our knowledge, the present study is the first to demonstrate that TsV and Ts1 can induce the production of inflammatory mediators by interacting with TLR2 and CD14/TLR4. We also demonstrated that the phosphorylation of NF-κB is TLR2- and TLR4/MyD88-dependent and is most likely mediated by Ts1, the primary component of TsV. In contrast, TLR2/MyD88-independent phosphorylation of c-Jun may be the result of other components of TsV. Although we observed the increased phosphorylation of c-Jun in TLR2^−/−^ and TLR4^−/−^ macrophages upon Ts1 stimulation, our primary findings are that venom recognition occurs through TLR2 and TLR4, resulting in NF-κB activation to generate IL-6 and TNF-α. Our results do not exclude the possibility that c-Jun activation might be due to other membrane receptor activation, which deserves further investigation.

These findings indicate that TsV signaling through TLR2 and TLR4/CD14 activates the NF-κB and MAPK signaling pathways, which may serve as important therapeutic targets for the prevention of the deleterious effects that result from the intense systemic inflammatory mediator release following *T. serrulatus* envenomation. Alternatively, although a number of antivenom treatments that typically attempt to neutralize the harmful venom exist, our results suggest that TLR antagonism may have potential therapeutic benefits following envenomation.

## Supporting Information

Figure S1 **TsV and Ts1 do not affect cell viability.** (A) Adherent macrophages were stimulated for 24 h with TsV at the indicated concentrations (25–150 µg/ml) in a 5% CO_2_ atmosphere at 37°C. Cell viability was measured using the MTT assay. Each column represents the mean value of 8 samples from 2 independent experiments. (B) Peritoneal macrophages were stimulated with TsV or Ts1 (50 µg/mL) for 24 h. After this period the disrupt membrane cell was determined by LDH release. *p<0.001 (one-way ANOVA) compared to the medium alone. (C and D) TsV or Ts1 inactivation by heating reduces IL-6 and TNF-α release in peritoneal macrophages. 50 µg/mL of TsV or Ts1 were heated or not at 60°C or 95°C and after 24 hours the cytokines were measured in the supernatant by ELISA. *p<0.001 (one-way ANOVA) compared to the medium alone; ^#^ *p*<0.001 compared to TsV room temperature (RT) and ^&^ *p*<0.001 compared to Ts1 RT.
(TIF)

Figure S2 **Peritoneal macrophages from C57Bl/6 mice stimulated with TsV and LPS (0.5 µg/ml) were pre-incubated with or without polymyxin B (poly) to neutralize contaminating LPS.** After 24 h, the amount of TNF-α in the supernatant was determined by ELISA. *p<0.001 (one-way ANOVA) compared to the medium alone (dashed line). The values are expressed as the mean ± SD (*n* = 8). The data are from 2 independent experiments.
(TIF)

Figure S3 **p38 and ERK1/2 MAPKs are involved in TsV-induced macrophage activation.** p38 (**a**), ERK1/2 (**b**) and JNK1/2 (**c**) expression was determined in peritoneal macrophages from C57Bl/6 mice stimulated with TsV (50 µg/ml) for 10, 30, 60 or 120 min. Medium alone was used as the negative control. The data are expressed as the fold-increase over the control (PBS) ± standard deviation (SD). *p<0.05 (one-way ANOVA) compared to medium alone (dashed line). The values are expressed as the mean ± SD (*n* = 4), and the data are from 2 independent experiments.
(TIF)

Figure S4 **TsV induces the phosphorylation of NF-κB, IκBα and c-Jun in stimulated macrophages.** Adherent peritoneal macrophages from WT (C57Bl/6) mice were stimulated with TsV (50 µg/ml) for 10 min, 2 and 24 h in a 5% CO_2_ atmosphere at 37°C. Medium alone was used as the negative control. p-NF-κB (**a**), p-IκBα (**b**) and p-c-Jun (**c**) protein levels were measured using the PathScan Inflammation Multi-Target Sandwich ELISA kit. The results are presented as the percentage of the relative levels of the phosphoproteins and are normalized to the positive control (100%). *p<0.05 (one-way ANOVA) compared to medium alone. The values are expressed as the mean ± SD (*n* = 4), and the data are from 2 independent experiments.
(TIF)
