# Supplementary figures and images for: TLR2, TLR4 and CD14 Recognize Venom-Associated Molecular Patterns from Tityus serrulatus to Induce Macrophage-Derived Inflammatory Mediators
Source: PLoS One. 2014 Feb 7;9(2):e88174. doi: 10.1371/journal.pone.0088174 (PMC3917877; doi:10.1371/journal.pone.0088174)

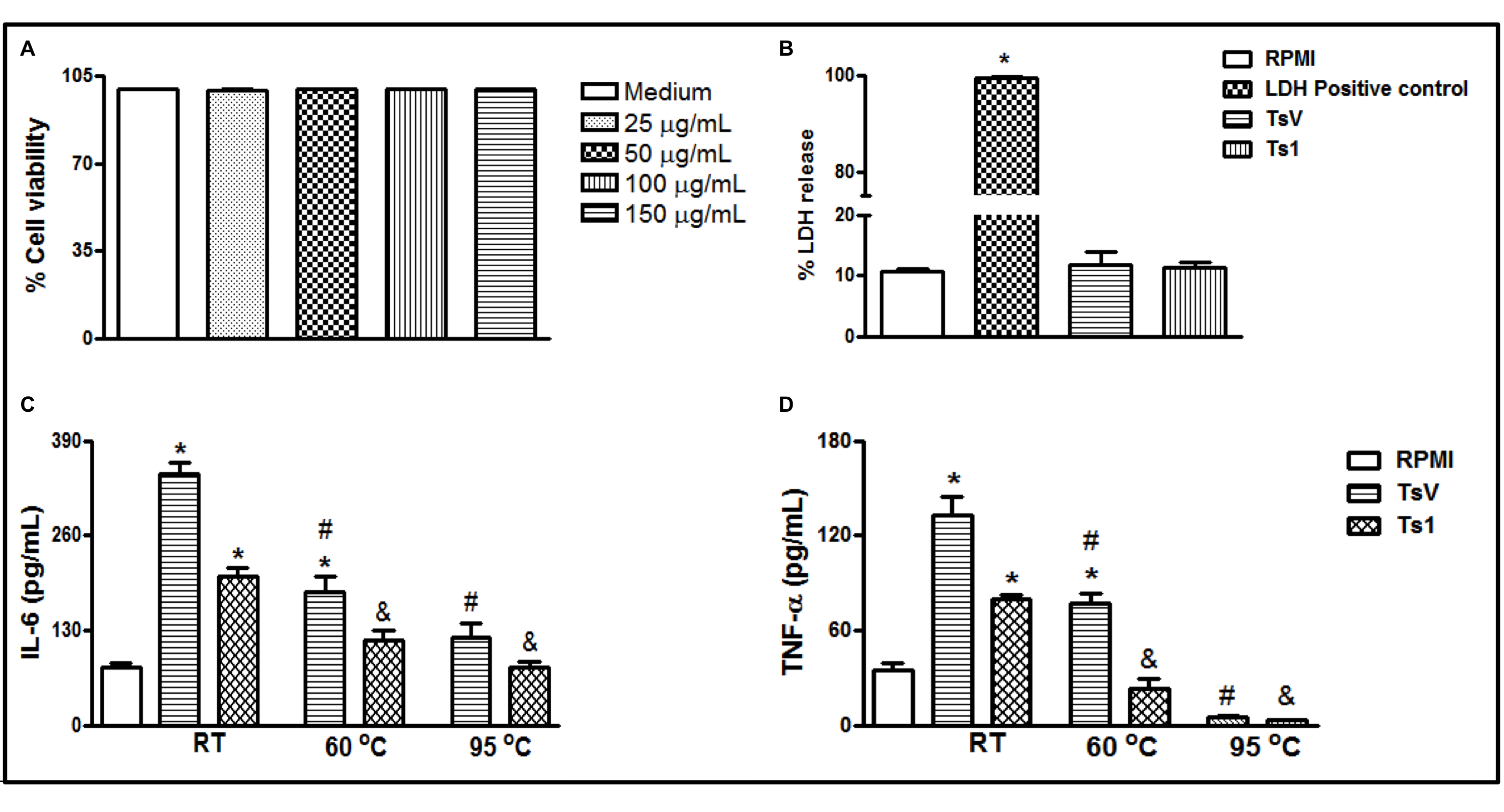

Supplement: Figure S1 — TsV and Ts1 do not affect cell viability. (A) Adherent macrophages were stimulated for 24 h with TsV at the indicated concentrations (25–150 µg/ml) in a 5% CO2 atmosphere at 37°C. Cell viability was measured using the MTT assay. Each column represents the mean value of 8 samples from 2 independent experiments. (B) Peritoneal macrophages were stimulated with TsV or Ts1 (50 µg/mL) for 24 h. After this period the disrupt membrane cell was determined by LDH release. *p<0.001 (one-way ANOVA) compared to the medium alone. (C and D) TsV or Ts1 inactivation by heating reduces IL-6 and TNF-α release in peritoneal macrophages. 50 µg/mL of TsV or Ts1 were heated or not at 60°C or 95°C and after 24 hours the cytokines were measured in the supernatant by ELISA. *p<0.001 (one-way ANOVA) compared to the medium alone; # p<0.001 compared to TsV room temperature (RT) and & p<0.001 compared to Ts1 RT. (TIF) [file pone.0088174.s001.tif]

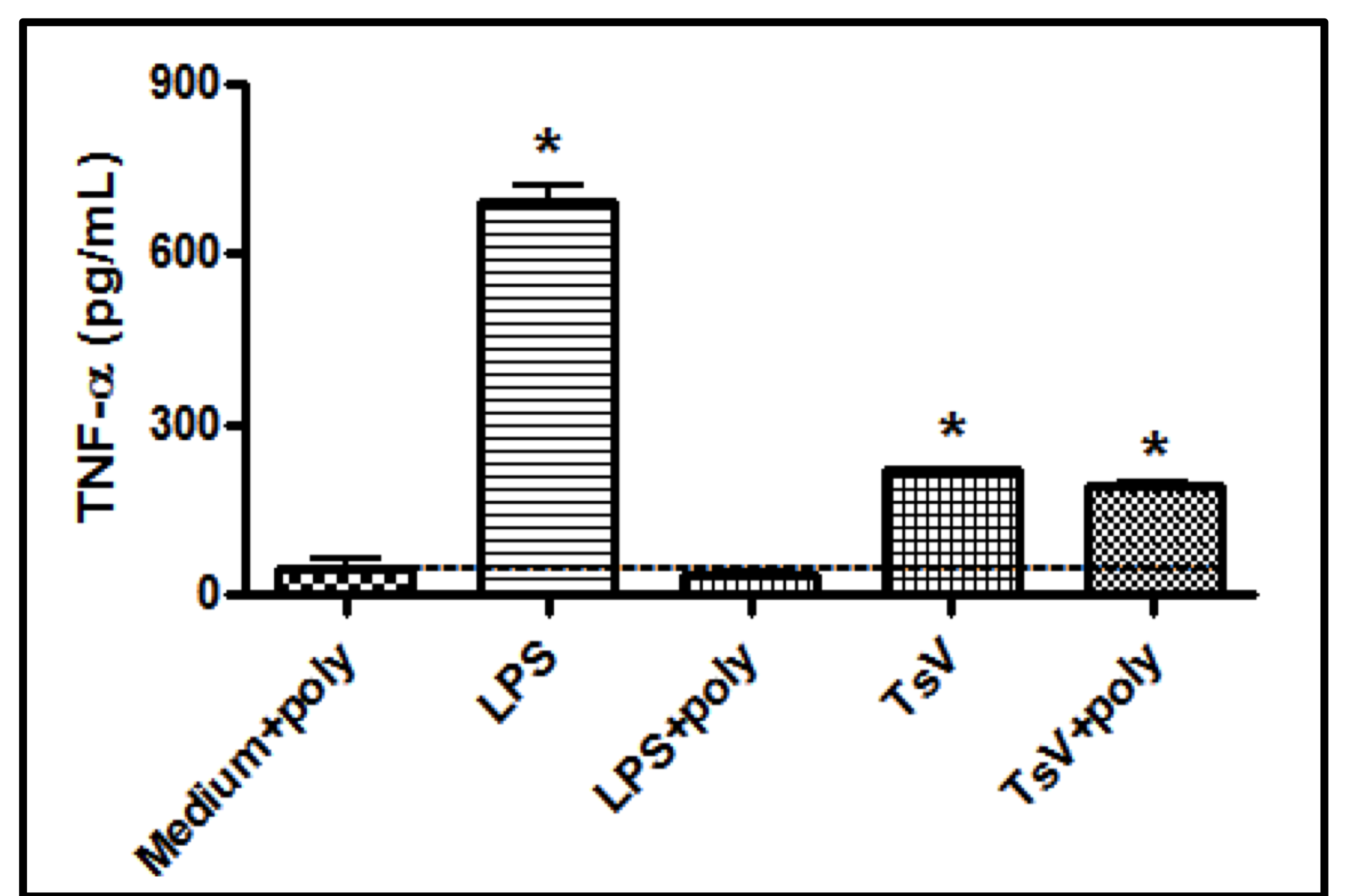

Supplement: Figure S2 — Peritoneal macrophages from C57Bl/6 mice stimulated with TsV and LPS (0.5 µg/ml) were pre-incubated with or without polymyxin B (poly) to neutralize contaminating LPS. After 24 h, the amount of TNF-α in the supernatant was determined by ELISA. *p<0.001 (one-way ANOVA) compared to the medium alone (dashed line). The values are expressed as the mean ± SD (n = 8). The data are from 2 independent experiments. (TIF) [file pone.0088174.s002.tif]

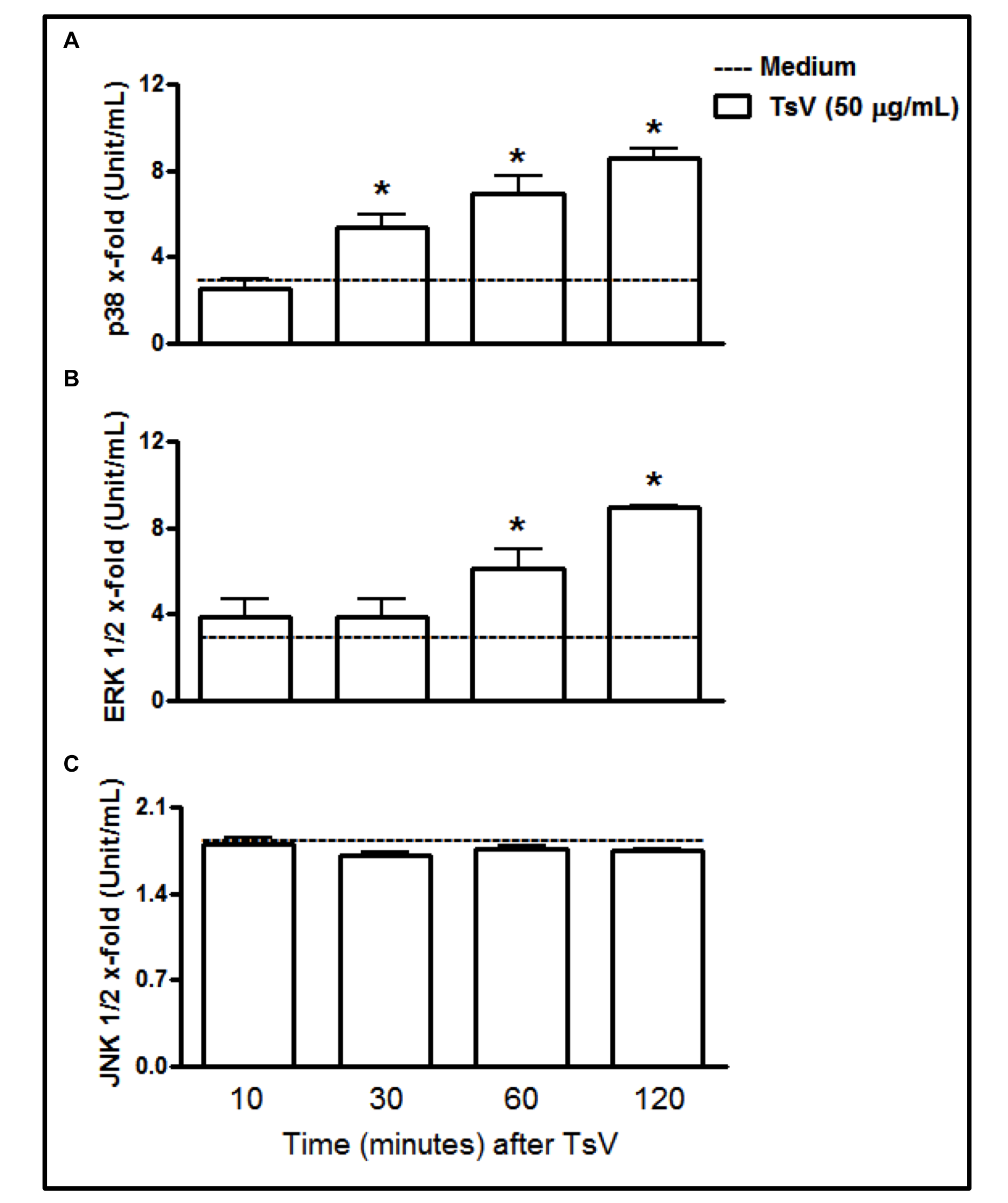

Supplement: Figure S3 — p38 and ERK1/2 MAPKs are involved in TsV-induced macrophage activation. p38 (a), ERK1/2 (b) and JNK1/2 (c) expression was determined in peritoneal macrophages from C57Bl/6 mice stimulated with TsV (50 µg/ml) for 10, 30, 60 or 120 min. Medium alone was used as the negative control. The data are expressed as the fold-increase over the control (PBS) ± standard deviation (SD). *p<0.05 (one-way ANOVA) compared to medium alone (dashed line). The values are expressed as the mean ± SD (n = 4), and the data are from 2 independent experiments. (TIF) [file pone.0088174.s003.tif]

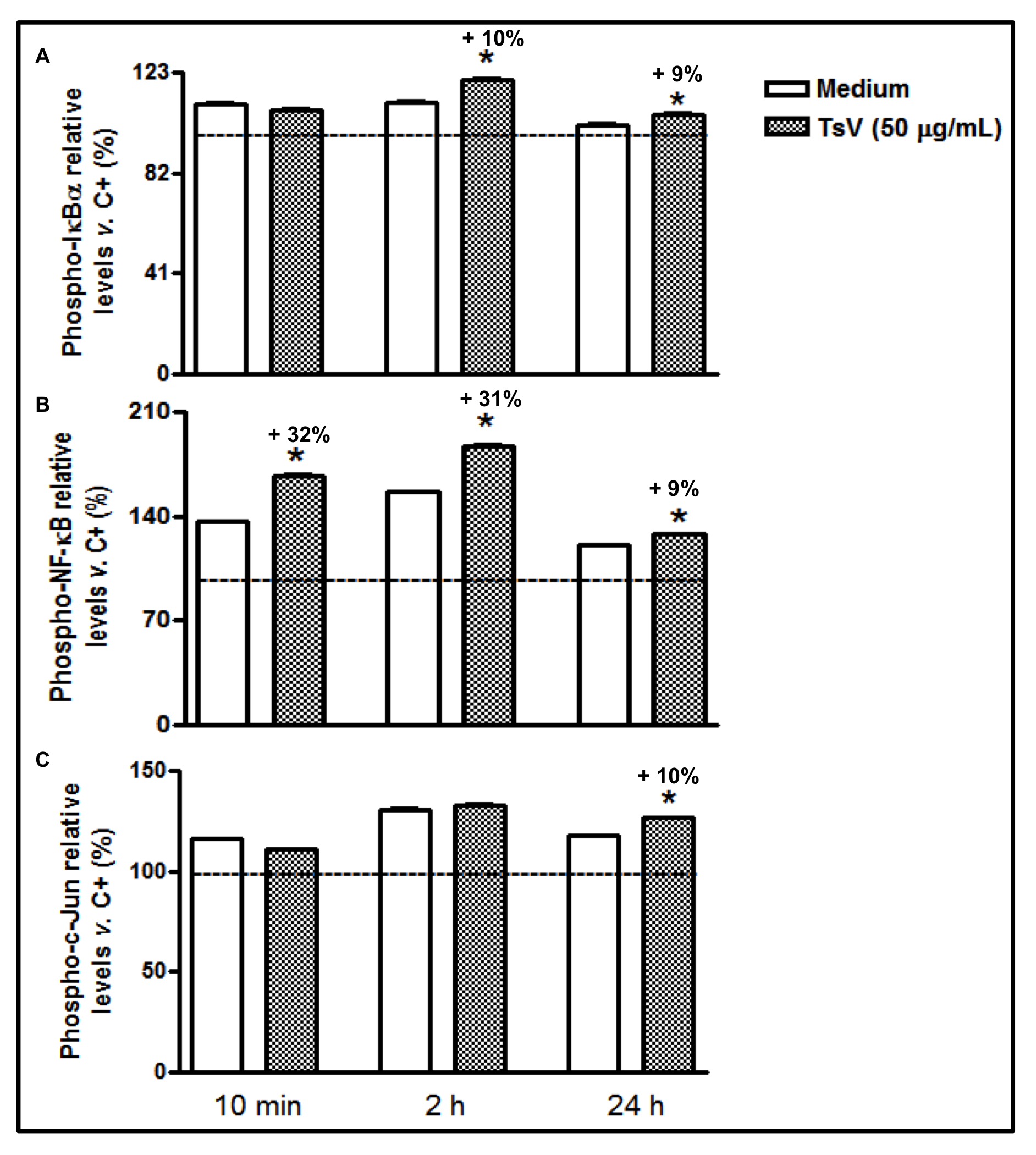

Supplement: Figure S4 — TsV induces the phosphorylation of NF-κB, IκBα and c-Jun in stimulated macrophages. Adherent peritoneal macrophages from WT (C57Bl/6) mice were stimulated with TsV (50 µg/ml) for 10 min, 2 and 24 h in a 5% CO2 atmosphere at 37°C. Medium alone was used as the negative control. p-NF-κB (a), p-IκBα (b) and p-c-Jun (c) protein levels were measured using the PathScan Inflammation Multi-Target Sandwich ELISA kit. The results are presented as the percentage of the relative levels of the phosphoproteins and are normalized to the positive control (100%). *p<0.05 (one-way ANOVA) compared to medium alone. The values are expressed as the mean ± SD (n = 4), and the data are from 2 independent experiments. (TIF) [file pone.0088174.s004.tif]
